# Letter to the editor: Potential causes of the decreased effectiveness of the influenza A(H1N1)pdm09 strain in live attenuated influenza vaccines

**DOI:** 10.2807/1560-7917.ES.2016.21.45.30394

**Published:** 2016-11-10

**Authors:** Christopher S Ambrose, Helen Bright, Raburn Mallory

**Affiliations:** 1 MedImmune, Gaithersburg, MD, United States; 2 MedImmune, Speke, United Kingdom

**Keywords:** influenza, vaccines and immunisations, live attenuated influenza vaccine, LAIV, vaccine effectiveness

**To the editor:** We greatly appreciate the editorial by Penttinen and Friede summarising the data regarding recent observations in the United States (US) of decreased effectiveness of the influenza A(H1N1)pdm09 strains (A/California/7/2009 and A/Bolivia/559/2013) included in live attenuated influenza vaccines (LAIV) [1]. Multiple hypotheses have been suggested as potential explanations for the reduced effectiveness compared with inactivated influenza vaccines (IIV). The most frequently cited hypotheses include poor replicative fitness of the A(H1N1)pdm09 LAIV strains, vaccine–virus interference in the quadrivalent formulation, reduced LAIV replication due to preexisting anti-influenza immunity from prior influenza vaccinations, and poor thermostability of A(H1N1)pdm09 LAIV strains. We have systematically evaluated each of these hypotheses and would like to share our assessments in case they might benefit ongoing international scientific discussions regarding LAIV effectiveness.

Based on evidence presently available to us, we believe that reduced replicative fitness of the A/California and A/Bolivia (H1N1)pdm09 LAIV strains is the most probable root cause for the reduced vaccine effectiveness (VE). From 2010/11 through 2013/14, LAIV VE in children aged 2–17 years against matched A(H3N2) and B strains has been comparable to that observed with IIV [2,3]. In 2014/15, LAIV4 VE against mismatched A(H3N2) strains was low, similar to that observed with IIV [2], and similar to that of LAIV3 against mismatched A(H3N2) strains that are ≥ 8-fold different by haemagglutination-inhibition assay [4,5]. Laboratory studies that we have conducted since April 2016 show that A/California and A/Bolivia strains have reduced replication in a human alveolar cell line and in primary human nasal epithelium air-liquid cultures, as well as reduced binding to α2,6-linked sialic acid receptors—the primary receptor for influenza viruses in the human upper respiratory tract. Consequently, we are actively working to identify a new A(H1N1)pdm09 LAIV strain with replicative fitness superior to that of A/California and A/Bolivia and similar to the replicative fitness of LAIV strains that previously demonstrated high levels of effectiveness in children.

In the context of reduced replicative fitness, vaccine–virus interference may have contributed to the observed reduced VE. However, vaccine–virus interference specific to the quadrivalent formulation appears to be an unlikely root cause of the reduced VE with LAIV. LAIV3 demonstrated reduced VE against A(H1N1)pdm09 in 2010/11 in the US [2] and 2012/13 in Germany [6]. Additionally, no VE was observed against A(H1N1)pdm09 strains in a randomised placebo-controlled study in children aged 2–5 years with trivalent A/Leningrad LAIV [7]. As reduced VE against A(H1N1)pdm09 strains was observed with trivalent LAIV formulations, any effects of vaccine–virus interference do not appear specific to the quadrivalent LAIV formulation.

Because rates of vaccine coverage in the US have historically been higher compared with European countries, questions have been raised regarding the role of prior vaccination in the reduced A(H1N1)pdm09 effectiveness [1]. Available data suggest that preexisting anti-influenza immunity due to prior vaccination is an unlikely root cause of the reduced VE observed with LAIV. In 2013/14 and 2015/16, the effect of prior season influenza vaccination on LAIV VE was evaluated in the US-based Centers for Disease Control and Prevention Flu VE and Influenza Clinical Investigation for Children (ICICLE) studies [8-10]. No statistically significant effect of prior season vaccination on LAIV VE was observed in either study in any season. Additionally, in the ICICLE study and in a large cohort study of children aged 24–35 months in Finland, most LAIV recipients were previously vaccinated. VE estimates trended higher among children vaccinated against influenza compared with unvaccinated children in the prior season in the ICICLE 2013/14 study (19% (95% confidence interval (CI): -80 to 64) vs 9% (95%CI: -161 to 68)); the ICICLE 2015/16 study (60% (95% CI: 1 to 84) vs 35% (95% CI: -206 to 86)), and the Finland study (74% (95% CI: 48 to 87) vs 25% (95% CI: -27 to 56)) [11].

In 2013/14, with LAIV4 containing the A/California strain, a statistically significant correlation was observed between reduced LAIV VE against (H1N1)pdm09 viruses and higher outdoor temperatures during LAIV lot unloading at US distributors [12]. In laboratory experiments, A/California demonstrated increased heat degradation [13], including experiments that simulated heat exposures that may have occurred during US distribution (33 °C for 4 hours). Environmental heat exposure has also been suggested as a contributing factor to the lack of LAIV VE against A(H1N1)pdm09 viruses in a randomised placebo-controlled study in children aged <5 years with trivalent A/Leningrad LAIV [14]. However, reduced VE was also observed in 2015/16 with A/Bolivia, the strain chosen to replace A/California based on its being more heat stable [1]. Consequently, although the reduced thermostability of A/California appears to have contributed to the low VE observed in 2013/14 in the US, it cannot explain the observations of reduced VE against A(H1N1)pdm09 strains in 2015/16 as A/Bolivia was thermostable.

We have initiated a multifaceted scientific investigation into the causes of the recently observed reduced effectiveness of LAIV, with the goal of identifying a more effective A(H1N1)pdm09 LAIV strain for potential inclusion in the 2017/18 LAIV formulation. All potential hypotheses continue to be evaluated. We welcome the input and support of the multiple stakeholders involved, including national public health agencies, the World Health Organization, and additional external scientific experts, as we work together to ensure that VE of LAIV is improved in future influenza seasons.
